# Mn Additive Improves Zr Grain Boundary Diffusion for Sintering of a Y-Doped BaZrO_3_ Proton Conductor

**DOI:** 10.1021/acsami.3c16359

**Published:** 2024-02-22

**Authors:** Su Jeong Heo, Steven P. Harvey, Andrew G. Norman, Muhammad Anisur Rahman, Prabhakar Singh, Andriy Zakutayev

**Affiliations:** † Materials Science Center, National Renewable Energy Laboratory, Golden, Colorado 80401, United States; ‡ Advanced Fuel Cycle Technology Development Division, Korea Atomic Energy Research Institute, 111 Daedeok-daero, Daejeon 34057, South Korea; § Department of Materials Science and Engineering, University of Connecticut, Storrs, Connecticut 06269, United States

**Keywords:** BZY, protonic conductor, intermediate temperature, electrochemistry, combinatorial thin films, pulsed laser deposition

## Abstract

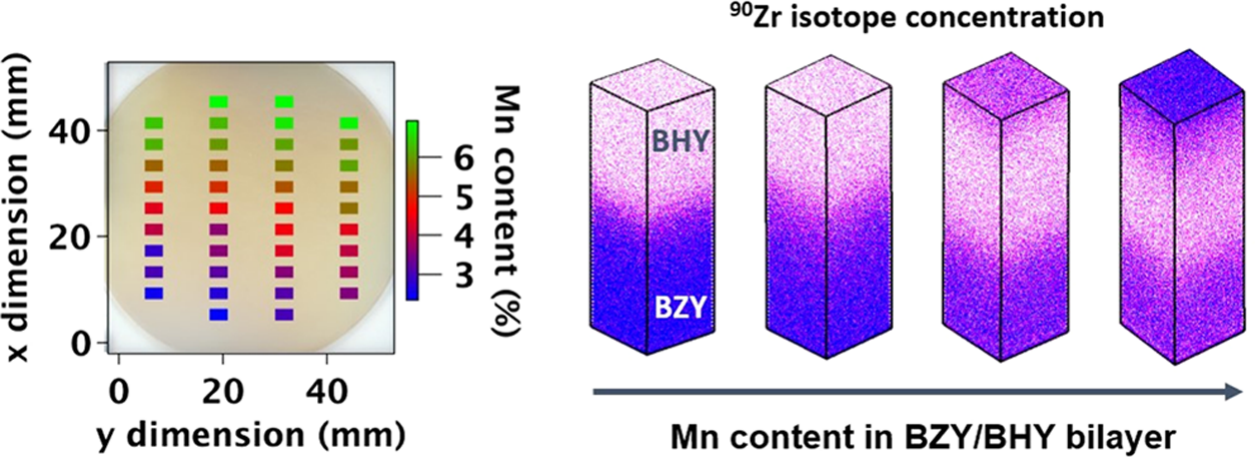

Yttrium-doped barium zirconate (BZY) has garnered attention as a protonic conductor in intermediate-temperature electrolysis and fuel cells due to its high bulk proton conductivity and excellent chemical stability. However, the performance of BZY can be further enhanced by reducing the concentration and resistance of grain boundaries. In this study, we investigate the impact of manganese (Mn) additives on the sinterability and proton conductivity of Y-doped BaZrO_3_ (BZY). By employing a combinatorial pulsed laser deposition (PLD) technique, we synthesized BZY thin films with varying Mn concentrations and sintering temperatures. Our results revealed a significant enhancement in sinterability as Mn concentrations increased, leading to larger grain sizes and lower grain boundary concentrations. These improvements can be attributed to the elevated grain boundary diffusion of zirconium (Zr) cations, which enhances material densification. We also observed a reduction in Goldschmidt’s tolerance factor with increased Mn substitution, which can improve proton transport. The high proton conduction of BZY with Mn additives in low-temperature and wet hydrogen environments makes it a promising candidate for protonic ceramic electrolysis cells and fuel cells. Our findings not only advance the understanding of Mn additives in BZY materials but also demonstrate a high-throughput combinatorial thin film approach to select additives for other perovskite materials with importance in mass and charge transport applications.

## 1 Introduction

Oxide materials with perovskite structure constitute a large and versatile class of compounds characterized by a general formula of ABO_3_, where numerous A and B metal elements can be accommodated.^1^ These materials exhibit a wide spectrum of electrical properties, from metallic or superconducting to semiconducting or insulating, thus rendering them subjects of extensive exploration across various applications.^2,3^ Notably, perovskite compounds doped with acceptor ions have gained substantial attention due to their efficacy as solid electrolytes in proton-conducting solid oxide electrolysis cells (H-SOECs)^4^ and solid oxide fuel cells (SOFCs),^5^ solar thermochemical hydrogen production (STCH),^6^ and chemical sensors.^7^ These electrochemical applications are enabled by the facile transport of protons within the perovskite lattice, which is characterized by low activation barriers for high proton conductivity.^8^ Moreover, the utilization of protons as opposed to oxygen ions for electrolyte conduction offers the distinct advantage of efficient fuel generation and utilization, with water production occurring at the “positrode” (anode in H-SOEC and cathode in SOFC)^9^ side of the device.^10^

Barium zirconate (BaZrO_3_) perovskite stands out as the most prevalent ceramic material employed as a proton-conducting electrolyte.^11^ It exhibits a higher-symmetry cubic structure when compared to distorted counterparts such as CaZrO_3_, SrZrO_3_, and BaCeO_3_.^12,13^ BaZrO_3_ is known for its remarkable mechanical strength and exceptional chemical stability, particularly when exposed to CO_2_ and H_2_O,^14^ thereby surpassing other candidate materials in these crucial aspects.^15^ Furthermore, when acceptor dopants like yttrium (Y) are introduced, BaZrO_3_ retained reasonably high proton conductivity, despite the accompanying lattice distortions and carrier localization.^16,17^ However, the total proton conductivity of Y-doped in BaZrO_3_ (BZY) is impeded by the significant grain boundary resistance that requires exceedingly high sintering temperatures (*T* > 1600 °C) and extended sintering duration (>24 h) to overcome the limitation.^18^ Consequently, reducing the sintering temperature while enhancing the proton conductivity of BZY is imperative to enhance its performance in electrochemical applications.

Aiming to refine the microstructure of BZY at lower temperatures, a range of sintering aids including ZrO, NiO, CuO, CoO, and FeO have been explored.^19−22^ For instance, the incorporation of a 4 mol % ZnO additive into BZY has yielded >93% relative densities at a lower sintering temperature (1300 °C for 4h), although the improvements in grain size and proton conductivity are modest. Similarly, the introduction of a 2 wt % NiO additive into BZY has induced the formation of BaY_2_NiO_5_ impurities that facilitate the sintering process. This, in turn, has promoted grain growth through partial decomposition at grain boundaries, ultimately leading to enhanced conductivity (3.3 × 10^–2^ S/cm) at 600 °C under a wet Ar atmosphere.^23^ Nevertheless, this method still demands high sintering temperature (1500 °C) and prolonged sintering duration (24 h). It is evident that the pursuit of alternative chemical additives is imperative to further alleviate the thermal demands of BZY sintering and bolster its proton conductivity. Notably, a recent study investigating Y and Mn co-substituted BaZrO_3_ has been published, highlighting Mn as a promising sintering aid for BZY.^24^

In this study, we present a novel approach where the incorporation of manganese (Mn) as an additive enhances the sintering of BZY proton conductors, surpassing the performance of other additives such as Zn and Ni. We have employed a combinatorial method to fabricate thin films, enabling us to systematically explore the influence of Mn. The smaller atomic radius of Mn compared to Zr results in a smaller lattice constant and a more ideal perovskite tolerance factor, as verified by X-ray diffraction (XRD) measurements. The presence of Mn-containing perovskites, characterized by a lower melting point, induces the development of an amorphous liquid-like phase within grain boundaries, leading to a remarkable reduction of 300–400 °C in sintering temperature and substantial grain growth, as confirmed by electron microscopy. To elucidate the function of the Mn additive in the BZY sintering process, we demonstrate that the activation energies for Zr elemental diffusion both in the bulk and along grain boundaries undergo a reversal when 6 atom % Mn is introduced in the sintering temperature of 900–1200 °C. This is evidenced through Zr/Hf depth profiles obtained via time-of-flight secondary ion mass spectroscopy (ToF-SIMS). Electrochemical impedance spectroscopy measurements reveal that the incorporation of the Mn additive in BZY sintered pressed pellets leads to a reduction of activation energies for grain boundary and total conductivity. This improvement manifests as enhanced proton conductivities at lower temperatures in a wet reducing atmosphere. In conclusion, these results imply that Mn has great potential as a sintering aid for BZY, promoting the expansion of its grain size and the improvement of its proton conductivity. Furthermore, this study highlights the benefits of using a high-throughput experimental approach for the discovery of additives in other ceramic materials.

## 2 Results

### 2.1 Grain Growth of BZY with the Mn Additive

**Figure 1:**
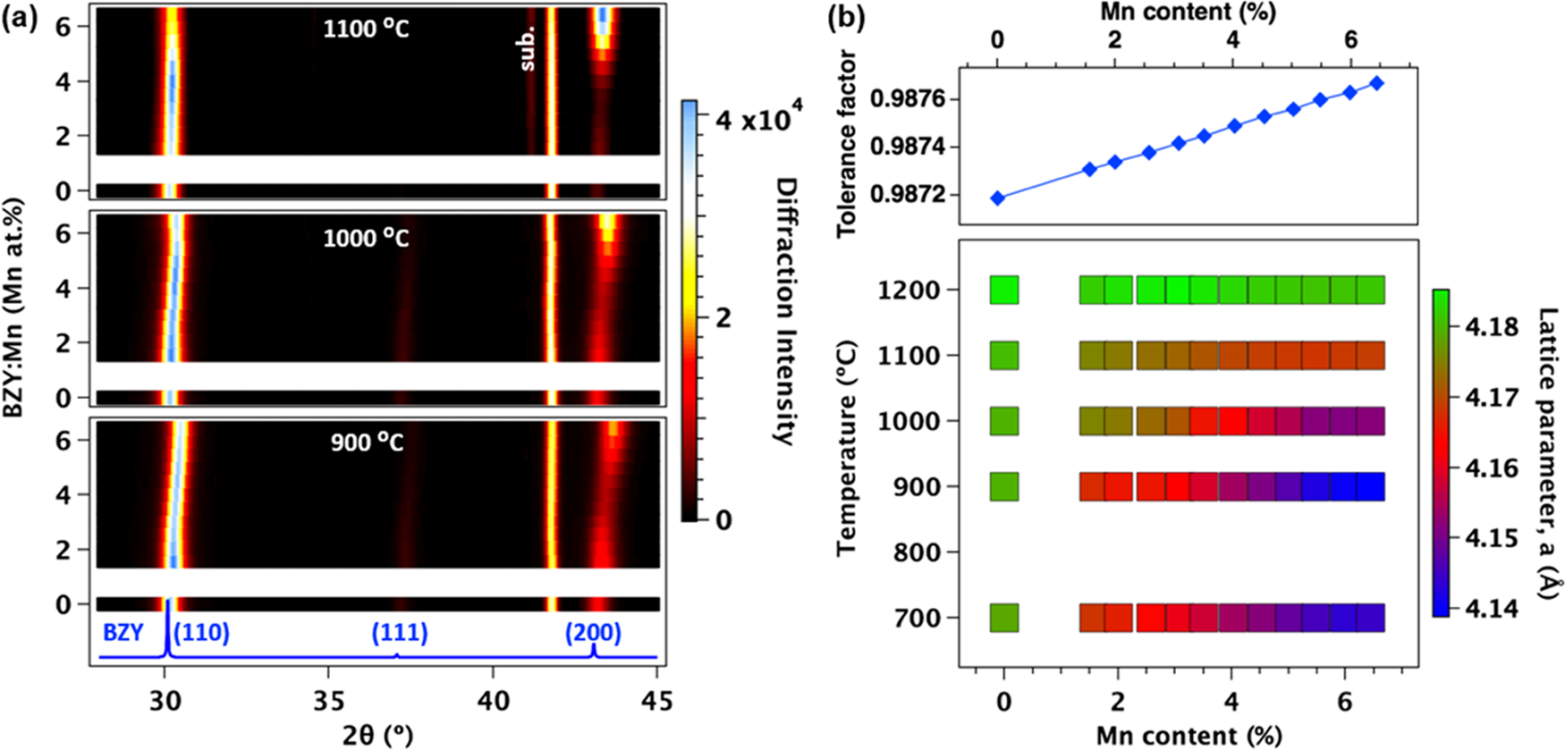
Crystal structure of BZY thin films as a function of Mn additive content and sintering temperature. (a) Color-scale map depicting intensity for θ–2θ XRD measurements following sintering at three different temperatures. (b) Graphical representation of the lattice parameter derived from the XRD results (bottom), along with the corresponding tolerance factors (top).

Combinatorial films of BZY and BZY with Mn additives were deposited onto 2 in. diameter sapphire (Al_2_O_3_) substrate heated to 700 °C, utilizing the combinatorial pulsed laser deposition (PLD) method. The Mn atomic ratio ranged from 0 to 6.5% to investigate its phase stability and grain size after sintering for 2 h at elevated temperatures (for detailed methods, refer to Section 5). Figure 1a displays color-scale maps of XRD patterns for the BZY:Mn compositionally graded films as a function of Mn content after sintering within the temperature range of 900–1100 °C.

In the absence of Mn, the BZY film exhibited a single phase with a cubic perovskite *Pm*3̅*m* structure. The major peaks at 30.1, 37.1, and 43.1° corresponded to (101), (111), and (200) crystal planes, respectively. Supplementary XRD patterns for the films within the temperature range of 700–1200 °C are provided in Figure S2.

Figure 1a illustrates a shift in the BZY reflection lines toward higher angles with increasing Mn substitution level up to a sintering temperature of 1100 °C. This shift indicates a reduction in the lattice parameter and unit cell volume. The decrease can be attributed to the smaller ionic radius of Mn^3+^ (0.64 Å), as expected from our PLD conditions,^25^ in comparison to Zr^4+^ (0.72 Å) in a 6-fold coordination. This reduction also leads to a more ideal perovskite tolerance factor closer to unity (Figure 1b). Conversely, at the highest sintering temperature (1200 °C), the Mn:BZY lattice parameter increases, accompanied by an elevated intensity ratio between (200) and (101) peaks compared to pure BZY. These variations might be associated with preferential growth along these crystalline planes or the formation of a Ba_2_AlO_4_ secondary phase due to the interaction of BZY with the Al_2_O_3_ substrate above 1100 °C.

**Figure 2:**
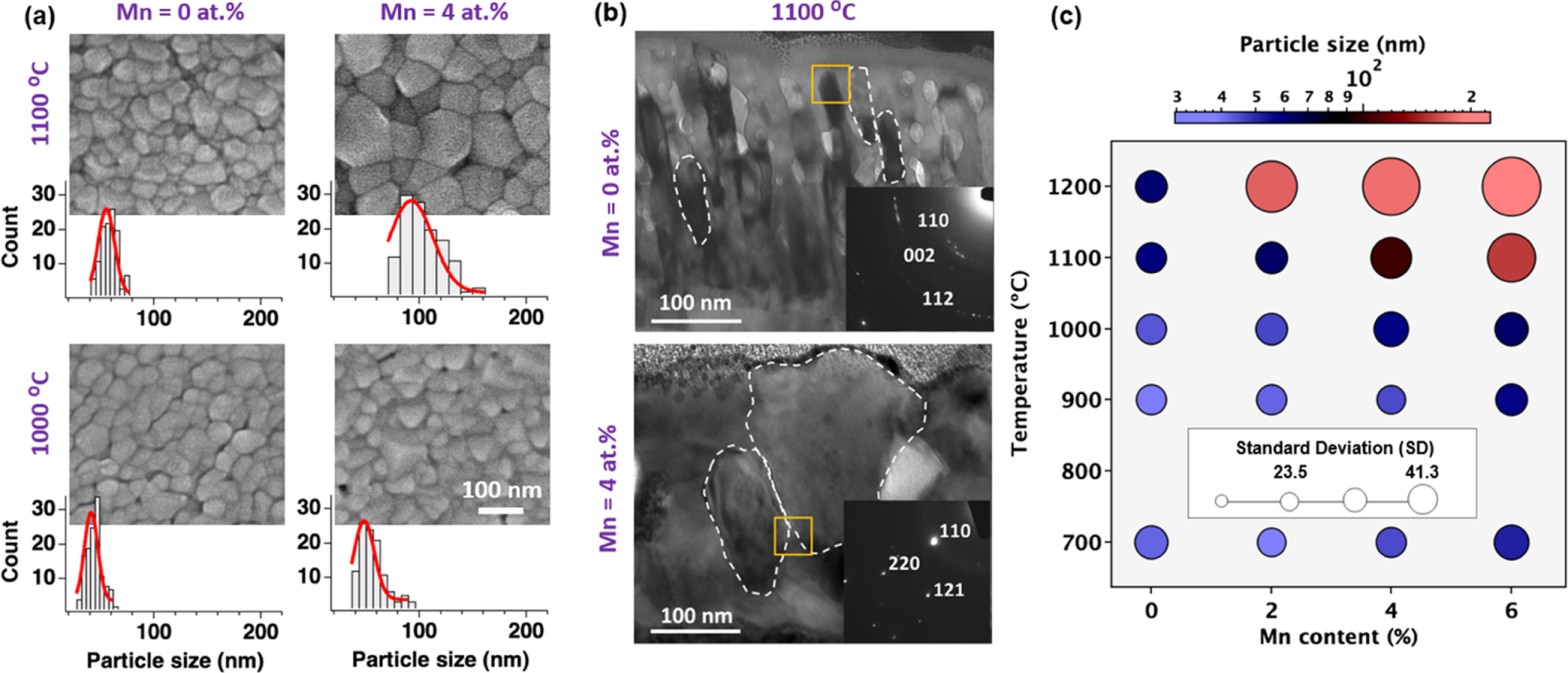
Surface and cross-sectional morphologies, and particle size of BZY and BZY:Mn thin films. (a) SEM surface micrographs of BZY and Mn:BZY (Mn = 4 atom %) thin films sintered at 1000 and 1100 °C. The distributions of grain sizes were inserted in each SEM images. (b) Cross-sectional TEM images of BZY and BZY:Mn (Mn = 4 atom %) films after sintering at 1100 °C. Insets display the SAED patterns corresponding to each layer. (c) Summary of average particle size dependent on Mn content and sintering temperature. The size of the circles corresponds to the standard deviation (SD) of the particle size distribution.

To assess the influence of Mn substitution on grain size, we examined the morphologies of BZY:Mn (Mn = 0–6.0 atom %) films following sintering at elevated temperatures, as illustrated in Figure 2a. The surface morphologies of as-deposited BZY and BZY:Mn films sintered up to 1000 °C revealed a dense microstructure with well-crystallized polyhedral grains. However, after sintering at 1100 °C or higher temperatures, the grain size of the BZY film exhibited minimal changes, while the grains in the BZY:Mn (Mn = 6 atom %) films noticeably increased in size. These results indicate that the introduction of Mn additives into BZY films enhances densification and sinterability. Grain size distributions were computed based on SEM images of all samples with varying Mn content and sintering temperatures (see Figure S3), and the results are presented in Figure 2c. Notably, the average grain sizes distinctly increased with higher Mn concentration, ranging from 61 nm for BZY to 192 nm for BZY with Mn = 6.0 atom % at 1200 °C.

To gain deeper insights into cross-sectional microstructures and particle size, we employed focused ion beam (FIB) technology to create lift-outs of BZY and BZY:Mn (Mn = 4 atom %) thin films sintered at 1100 °C. Subsequently, we collected bright-field transmission electron microscopy (TEM) images. The cross section of the BZY film (Figure 2b, top) displayed columnar grain boundaries with a grain size ranging from 50 to 100 nm, consistent with the plan-view SEM results (Figure 2a, top). The selected area electron diffraction (SAED), inset in Figure 2b, exhibited polycrystalline spots indexed to the cubic crystal system of the *Pm*3̅*m* space group along the [11̅0] direction. In contrast, the BZY:Mn film (Figure 2b, bottom) exhibited distinctive crystal growth with a grain size of 100–200 nm, also consistent with the SEM results (Figure 2a). The SAED pattern inserted in Figure 2b displayed fewer peaks that can be identified as the cubic perovskite structure (*Pm*3̅*m*) with the [11̅1] direction.

### 2.2 Elemental Diffusion in BZY with Mn Additive

To unravel the underlying mechanism responsible for the observed grain growth in BZY with Mn addition, we investigated the Mn:BYZ diffusion coefficients and activation energies for Zr elemental diffusion both within the bulk and along the grain boundaries. Typically, sintering studies for bulk powder involve geometric measurements of sintering shrinkage rate,^26,27^ which are impractical for much thinner thin films. Therefore, we turned to secondary ion mass spectrometry (SIMS) measurements on BZY|BHY and BZY:Mn|BHY:Mn film diffusion couples synthesized via combinatorial PLD. We selected the BHY:Mn layer as a reference because Hafnium (Hf) has a similar charge and ionic radius with Zr (0.71 Å vs 0.72 Å), and thus it is an apt tracer element that does not require isotopic studies. We chose to study B-site cations for diffusion because the literature suggests that B-size diffusion is slower than A-site diffusion in AZrO_3_ (A = Ca, Sr, Ba) and related perovskites,^28−31^ ultimately defining higher activation energy and influencing high sintering temperatures.

**Figure 3:**
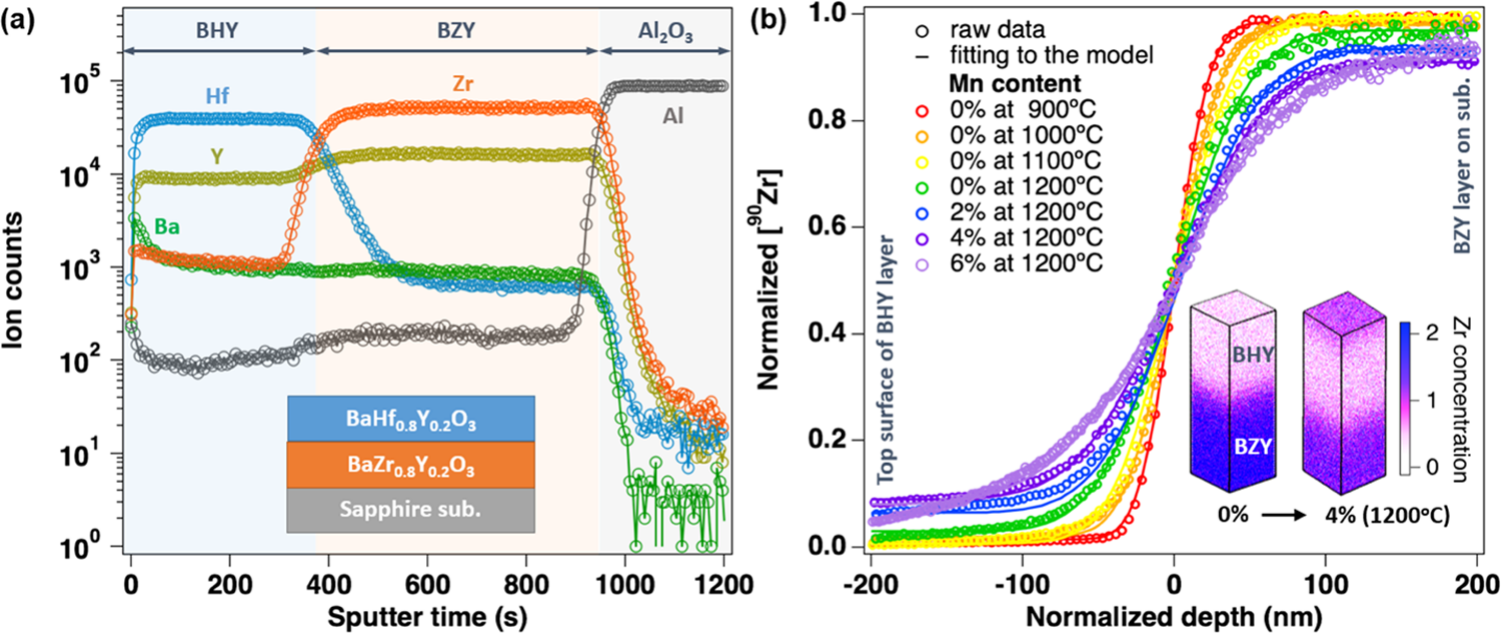
Composition depth profiles of BHY|BZY bilayers. (a) Representative depiction of ToF-SIMS results illustrating various species found in the as-deposited (at 700 °C) BHY|BZY bilayer, displayed in the inset. (b) Overview of normalized Zr fraction depth profile for Mn-free BHY|BZY bilayers sintered at 900–1200 °C and BHY|BZY bilayers featuring diverse Mn content, all sintered at 1200 °C. The 3D images inset in (b) show the Zr concentration distribution map for the BHY|BZY and BHY|BZY with 4% Mn at 1200 °C, respectively.

As depicted in Figure 3a, typical SIMS depth profiles illustrate the different species contained in the as-deposited BHY|BZY bilayer, with Figure S5 showing the profiles after sintering at 900–1200 °C in comparison to the BHY:Mn|BZY:Mn bilayers after sintering at the same temperature range. Figure S5 clearly highlights multilayer interdiffusion between BHY and BZY layers with the addition of Mn sintering aid at elevated sintering temperatures. In contrast, negligible interdiffusion between the BHY and BZY layers was observed without Mn additive at high sintering temperatures. These SIMS results support that Zr diffusion serves as a reliable indicator of the BZY sinterability.

Figure 3b compiles the Zr diffusion profiles for the Mn-free BZY sintered at 900–1200 °C and for Mn:BZY bilayers with Mn content ranging from 0 to 6 atom % sintered at 1200 °C. The fitted model curves closely align with the actual normalized profiles. Notably, the BZY profiles reveal that elevating the sintering temperature enhances Zr cation diffusion by shallowing the diffusion gradient, indicative of increased diffusion length. The Zr diffusion length, denoted as √*Dt*, characterizes the decay of the diffusion concentration with depth.^32^ As depicted in Figure S6, higher sintering temperatures and greater Mn content in BZY result in increased Zr diffusion length. Consequently, the profiles in Figure 3b indicate increased near-surface decay and deep-penetrating Zr diffusion tails at the interface at higher Mn content in BZY sintered at 1200 °C with a higher Zr concentration in the BHY layer compared to Mn-free BZY. The 3D Zr distribution maps (Figure 3b inset) provide clear visual evidence of faster Zr diffusion into the BHY layer in 4% Mn:BZY compared to Mn-free BZY.

**Figure 4:**
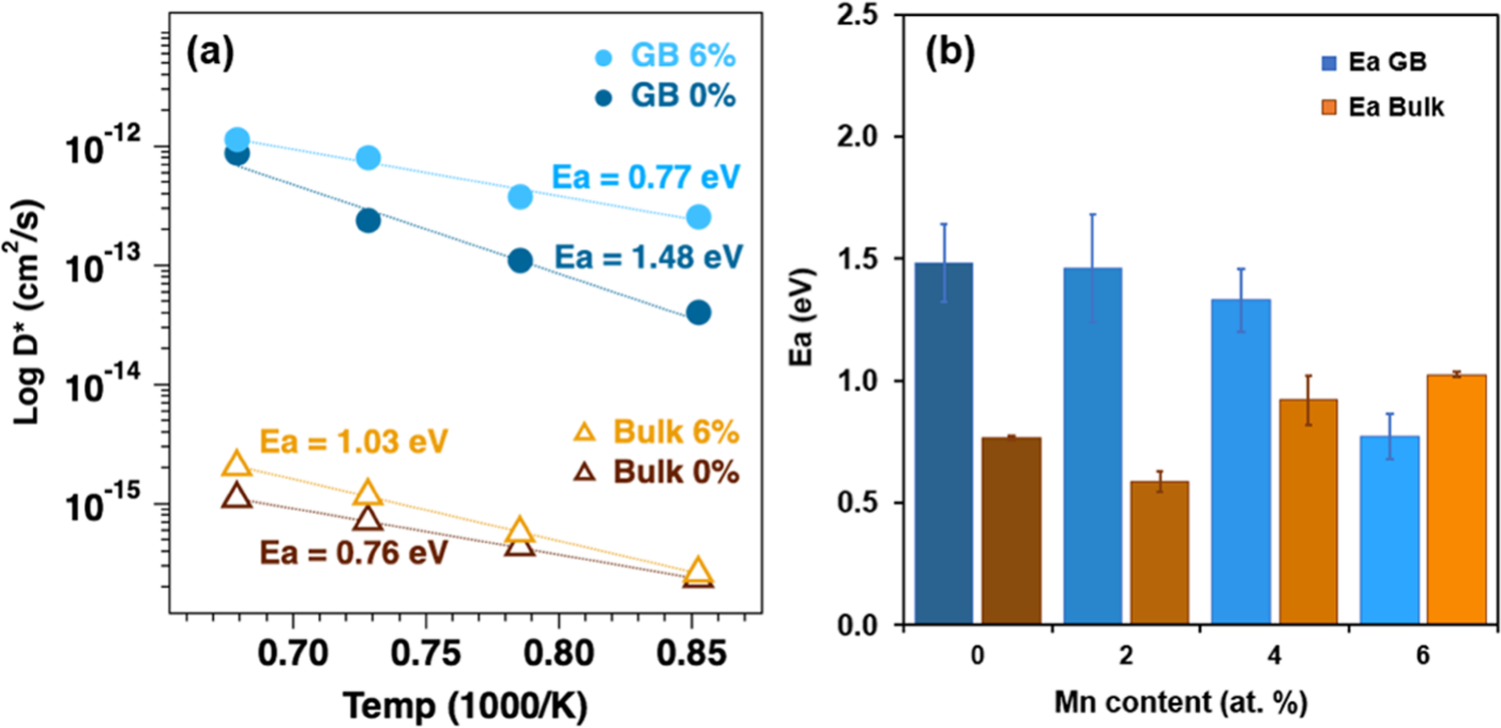
Zr cation diffusion properties of the BZY and Mn:BZY films. (a) Arrhenius plots illustrating the diffusion coefficients for Zr within the bulk and along grain boundaries, derived from Zr concentration profiles obtained via ToF-SIMS. (b) Activation energy (*E*_a_) for Zr diffusion in both bulk (*D*_b_) and grain boundaries (*D*_gb_) as a function of the Mn content in BZY.

Figure 4a displays diffusion coefficients as a function of the reciprocal temperature for Zr diffusion within the bulk and along the grain boundary. These coefficients were determined based on Zr concentration profiles derived from the BZY and Mn:BZY films. It is noteworthy that the grain boundary coefficients (*D*_gb_) are nearly 3 orders of magnitude higher than the bulk diffusion coefficients (*D*_b_) for Zr diffusion. For the entire range of Mn content examined within the 900–1200 °C temperature range (detailed in Figure S8), each of the *D*_b_ and *D*_gb_ values for Mn:BZY surpasses the corresponding values for pure BZY. To further elucidate these findings, bulk Zr diffusion lengths (√(*D*_b_*t*)) for all samples were juxtaposed with the particle sizes of BZY, resulting in the determination of Harrison’s classification.^33^ As illustrated in Figure S6, the effective Zr bulk diffusion lengths (ranging from 5 to 20 nm) are notably smaller (5 to 10 times) than the sizes of BZY particles (ranging from 40 to 200 nm). Consequently, our estimation remains valid for both grain and grain boundary Zr diffusion.

Figure 4b show activation energies for Zr diffusion in both bulk and along grain boundaries, as extracted from Arrhenius fits of ln(*D*) vs 1/*T*. The corresponding pre-exponential factors are detailed in Figure S9. Notably, for Mn-free BZY, grain boundaries exhibit higher activation energy values (1.48 eV) compared to the bulk (0.76 eV). Additionally, the pre-exponential factors (*D*_0_) for grain boundaries are larger than those for the bulk, resulting in an overall higher Zr diffusion coefficient for grain boundaries when compared to the bulk. However, as the Mn content increases, an interesting trend emerges: the activation energy for Zr diffusion along grain boundaries decreases, while the activation energy for Zr diffusion in the bulk increases. This trend continues until grain boundaries are no longer the primary limiting factor for Zr diffusion. The parameter β, calculated as , serves as an indicator of the magnitude of grain boundary diffusivity relative to the bulk. β values exceeding 10 indicate that the grain boundary diffusivity is suitable.^34^Figure S10 illustrates that BZY with 6 atom % Mn sintered at 1200 °C fails to meet the β criteria (β > 10), suggesting that the grain boundary diffusion model is no longer suitable. This shift is likely attributed to the smallest grain boundary concentration and the largest grain size among all of the measured samples. Overall, these findings highlight the beneficial impact of the Mn additive on the sintering of the Y-doped BaZrO_3_ proton conductor, primarily by enhancing Zr diffusion along grain boundaries.

## 3 Discussion

### 3.1 Mechanism of Grain Growth with Mn Additive

At first glance, the observation that Zr diffusion lengths along grain boundaries are 2–3 orders of magnitude higher than that in the bulk (Figure 4a), yet the corresponding activation energies of Zr diffusion in the bulk are up to 2 times lower compared to grain boundaries (Figure 4b), may appear surprising. However, this counterintuitive difference observed here can be explained by the significantly higher concentration of special atomic sites in more defective grain boundaries, where defect-assisted Zr diffusion can occur. This is mathematically reflected in the 4–5 orders of magnitude higher grain boundary pre-exponential factor (Figure S9). This explanation, although different from conventional wisdom, aligns with prior literature reports on BaZrO_3_ powders where a similar 4-order of magnitude difference was observed.^28^ This challenges the conventional notion that higher diffusion lengths result from lower activation energies.

It is also noteworthy that the magnitude of activation energies of Zr diffusion in BZY films within the 900–1200 °C range (e.g., *E*_a_ = 0.76 eV for *D*_b_, 1.48 eV for *D*_gb_) reported here is substantially lower than that for BaZrO_3_ powder within the 1300–1500 °C range (*E*_a_ = 4.5 eV for *D*_b_, 3.7 eV for *D*_gb_) reported the literature.^28^ These differences significantly exceed the measurement uncertainties of activation energies, as indicated by relatively larger error bars in Figure 4b, particularly due to the anomalously high Zr diffusion observed at 1200 °C for all studied Mn concentrations. This dissimilarity in the magnitude of activation energies for Zr diffusion in BZY stems from the fact that the activation energy (3.7–4.5 eV) at high powder temperature encompasses both the defect formation energy and the migration energy, whereas the activation energy (0.76–1.48 eV) at low film temperature, where defect formation is frozen, represents only the migration energy of the cation diffusion.^35^

**Figure 5:**
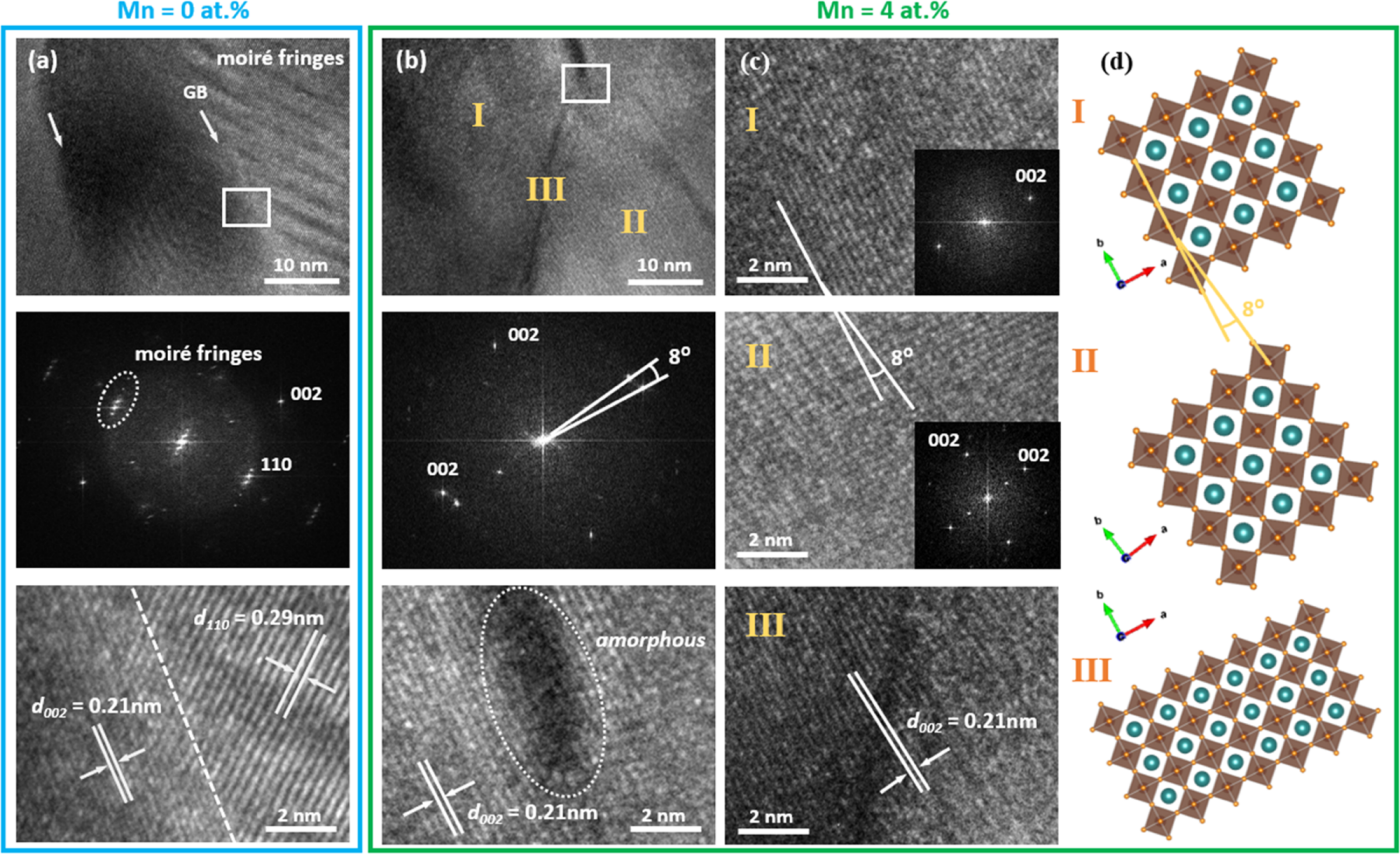
TEM images depicting the grain boundaries of BZY (Mn = 0%) and BZY:Mn (Mn = 4%) films sintered at 1100 °C. The images include bright-field images (top), FFT patterns (middle), and HRTEM images (bottom) obtained from marked areas in (a) BZY and (b) BZY:Mn (Mn = 4%) films, respectively. (c) HRTEM images of the region shown in (b) depicting lattice fringes between two grains (I and II) and grain boundaries (III), along with the corresponding atomic model (d).

The improved sinterability of BZY with Mn additive reported in this study can be attributed to the initiation of partial melting at grain boundaries, as observed by high-resolution TEM (HRTEM) analysis. Figure 5 illustrates TEM images for representative grain boundary regions of BZY and 4% Mn-BZY films after sintering at 1100 °C taken from the orange box in Figure 2b, with additional grain boundary regions shown in Figure S11. In the case of pure BZY, the grains surrounding the grain boundary exhibit several atomic planes, resulting in Moiré fringes rather than clear BZY lattice fringes (Figure 5a), indicating inefficient sintering. The HRTEM image in Figure 5a reveals lattice spacing of 0.21 nm corresponding to the (002) planes and 0.29 nm corresponding to the (110) planes of BZY. In contrast, the Mn:BZY sample exhibits no Moiré fringe pattern (Figure 5b), suggesting that the amorphous phase at this triple grain boundary (Figure 5b, bottom) displays “liquid-like” behavior that facilitates sintering. The HRTEM images of the three points (I–III) in Figure 5c reveal a slight lattice fringe mismatch (grains of I and II), resulting in a low-angle (8°) grain boundary, further illustrating the beneficial effect of the Mn additive on BZY grain sintering and crystal growth. Consequently, as sintering temperatures increase, grain boundaries increasingly exhibit “liquid-like” behavior (Figure 5), leading to enhanced Zr diffusion along grain boundaries (Figure 4) and substantial sintering and growth of the grains (Figure 2).

### 3.2 Comparison of Mn to Other Additives

One compelling reason for the improved sintering of BZY with the Mn additive is the lower melting point of Mn-containing perovskites compared to Y-doped BaZrO_3_. Most of the other studied additives have significantly higher melting points (1975 °C for ZnO, 1955 °C for NiO, 1377 °C for FeO, 1124 °C for Cu_2_O compared to 2715 °C for ZrO_2_ and 2430 °C for Y_2_O_3_),^36−38^ when compared to Mn_2_O_3_ (1244 °C) and/or Mn_3_O_4_ (940 °C) under our PLD conditions.^25^ Furthermore, these other additives are reported to form lower eutectic points with BaO (890 °C for BaO·CuO, 1099 °C for BaO·ZnO, 1080 °C for BaO·NiO, 1112 °C for BaO·CoO)^39−41^ compared to BaO·Y_2_O_3_ (1760 °C) and BaO·ZrO_2_ (2600–2700 °C).^42^ It is expected that the eutectic temperatures of BaO·Mn_2_O_3_ or BaO·Mn_3_O_4_ would be even lower.

Another likely beneficial effect of the Mn additive on the sintering of Y-doped BaZrO_3_ is the more ideal Goldschmidt’s tolerance factor of Mn:BZY, which also significantly enhances ionic conductivity.^43^ BaZrO_3_ possesses a high-symmetry cubic structure with ideal Zr–O and Ba–O bond lengths, resulting in a tolerance factor of 1.004. This is in contrast to other proton-conducting perovskite oxides such as CaZrO_3_ (*t* = 0.914), SrZrO_3_ (*t* = 0.947), and BaCeO_3_ (*t* = 0.943).^12,13^ However, the introduction of Y^3+^ (*r* = 0.90 Å for 6-fold coordination) as a dopant reduces the tolerance factor down to 0.987 at 20% Y-doped BZO, leading to local distortions that can impede proton transport.^17^ The sintering additives studied in the literature, with an ionic radius larger than Zr^4+^ (*r*^VI^ = 0.72 Å), such as Zn^2+^ (*r*^VI^ = 0.74 Å), Cu^2+^ (*r*^VI^ = 0.73 Å), Ho^3+^ (*r*^VI^ = 0.90 Å), or Ce^4+^ (*r*^VI^ = 0.87 Å),^22,44,45^ are likely to have a detrimental impact on proton conductivity by reducing the tolerance factor even further. In contrast, the proposition of the smaller Mn^3+^ ion (*r*^VI^ = 0.64 Å), confirmed through our prior study,^25^ has the potential to restore the BZY tolerance factor toward unity (Figure 1b). This, in turn, reduces local distortion and improves proton transport.

**Figure 6:**
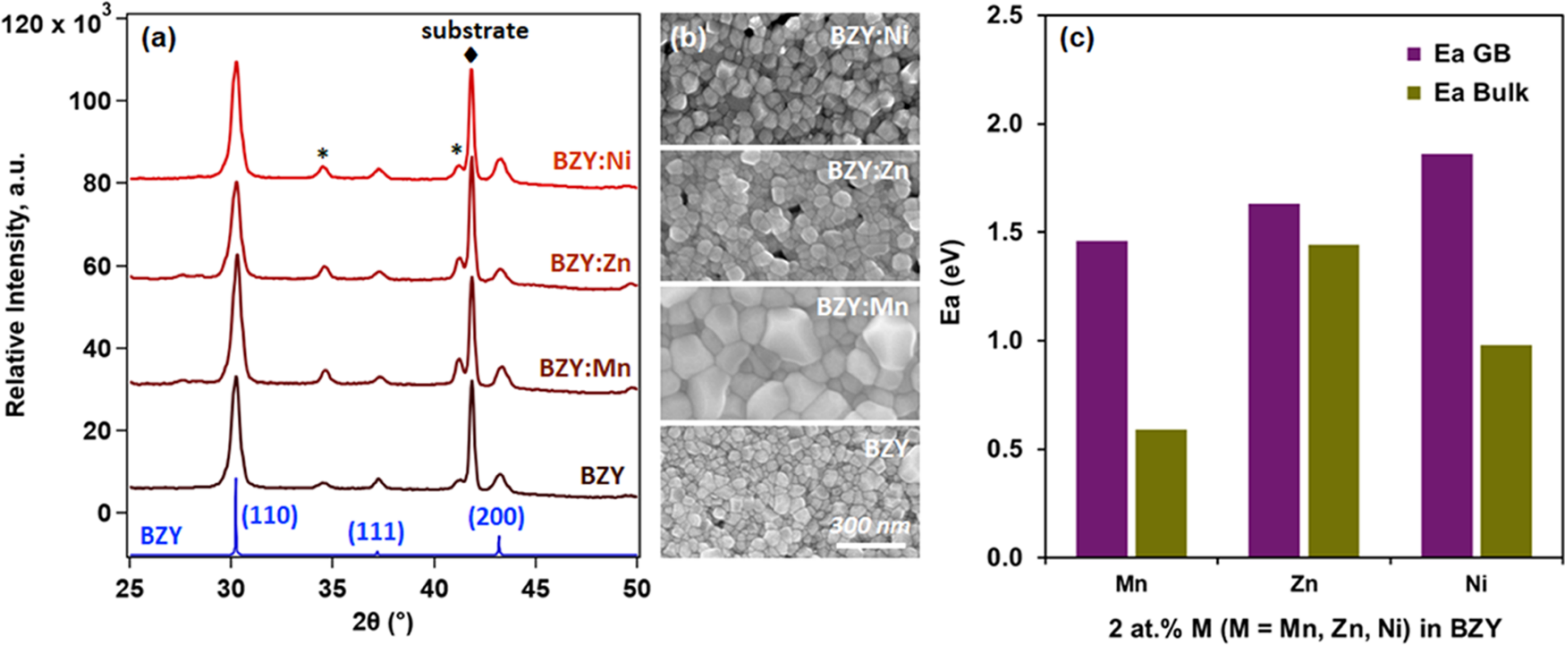
Comparative analysis of additives. (a) XRD patterns and (b) SEM surface morphologies of BZY and BZY thin films with 2 atom % additives of Mn, Zn, and Ni, sintered at 1200 °C. The potential presence of the BaAl2O_3_ phase is denoted by (*) in the XRD pattern. (c) Activation energies of Zr diffusion in bulk and grain boundaries, focusing on BZY thin films with 2 atom % additives of Mn, Zn, and Ni. All other experimental conditions remain consistent across the comparisons.

In the extended investigation, BZY thin films were fabricated with Zn and Ni additions using the same pulsed laser deposition (PLD) conditions applied to Mn-doped BZY, followed by annealing at 1200 °C for 2 h on a sapphire substrate. Figure 6 illustrates the outcomes of the comparative analysis. Despite exhibiting identical cubic perovskite *Pm*3̅*m* structures in XRD patterns, SEM surface morphologies distinctly reveal significant particle growth in Mn-added BZY as opposed to Zn and Ni additions.

The findings presented in this expanded section underscore the positive impact of Mn additives on particle growth, contrasting with the behavior observed with other additives investigated herein (Figure 6b). Additionally, our measurements (Figure 6c) disclose that Zn and Ni additives in BZY exhibit higher activation energies for Zr diffusion in both bulk (1.44 and 0.98 eV, respectively) and along grain boundaries (1.63 and 1.86 eV, respectively) compared to their Mn additive counterpart (*E*_a_ = 0.59 eV for *D*_b_, 1.46 eV for *D*_gb_). These measurements were conducted under consistent experimental conditions, encompassing the low sintering temperature range of 900–1200 °C and a 2-h sintering duration.

### 3.3 Proton Conductivity of BZY Pressed Pellets with Mn and Other Additives

**Figure 7:**
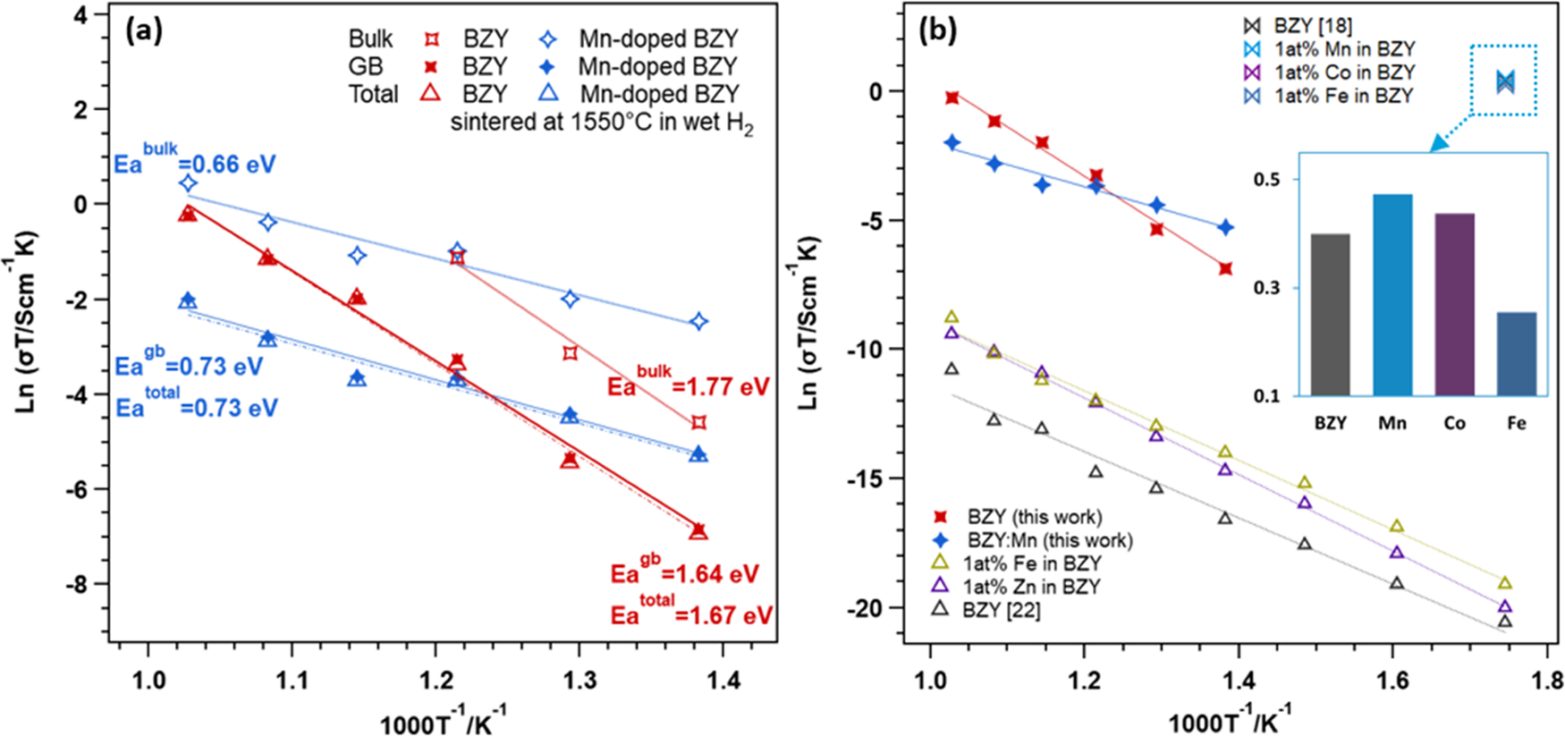
Conductivities of BZY and BZY with additives. (a) Arrhenius plots of bulk, grain boundary, and total conductivities in wet H_2_ (*p*H_2_O = 0.05 atm) of BZY (red) and BZY:Mn (blue) pressed pellets sintered at 1550 °C for 15 h. (b) Comparison of effective grain boundary conductivities of BZY and BZY:Mn, as measured in this study, with those of BZY and BZY with Fe, Zn additives^22^ and those of BZY and BZY with Mn, Co, and Fe additives^18^ for proton conduction reported in the literature.

An important factor in determining the future applications of the Mn:BZY is the proton transport behavior of the BZY with Mn additives. To assess the effect of the Mn additive on BZY proton conduction, we prepared BaZr_0.8_Y_0.2_O_3−δ_ (BZY) and BZY with Mn = 2.0 wt % (BZY:Mn) pressed pellets sintered at 1550 °C for 15 h, conditions favorable for pure BZY. We then measured their conductivities in wet H_2_ (*p*H_2_O = 0.05 atm) at intermediate temperatures (400–700 °C). The microstructures of the samples after sintering and representative impedance spectra for BZY and BZY:Mn measured at 500 and 600 °C in wet H_2_ are shown in Figure S12. By analyzing the electrochemical impedance spectra, we separated contributions from bulk and grain boundary conduction mechanisms and determined the total conductivities as their sum,^46,47^ as depicted in the Arrhenius plots in Figure 7a. In the temperature range of 600–700 °C, both effective grain boundary and total conductivities of BZY:Mn are lower than those of BZY. However, at lower temperatures (400–500 °C), the conductivities of BZY:Mn are higher than those of BZY due to lower activation energies of BZY:Mn and higher grain boundary resistance of BZY. Notably, the grain boundary plays a significant role in decreasing the operating temperature of electrolysis cells, as its contribution increases at reduced temperatures.^48^

The effective grain boundary conductivities of BZY and BZY:Mn measured in this study were also compared with data from the literature for other additives, as shown in Figure 7b. The effective grain boundary conductivities of BZY and BZY with Fe and Zn additives, sintered at 1400 °C for 6 h and measured in dry hydrogen,^22^ were found to be lower than those of BZY and BZY:Mn in this study. In that literature report, the Fe and Zn additives in BZY improved its effective grain boundary conductivity, but it led to steeper slopes in the Arrhenius plot representing the activation energies. The enlarged box in Figure 7b shows an increase in proton conductivity along the grain boundary of bulk BZY with Mn compared to the Co and Fe additives. For example, at 300 °C in a wet H_2_ atmosphere, the proton conductivities of 1 atom % Mn-BZY along the grain boundaries surpassed those of bare BZY and other additives such as Co and Fe,^18^ which is consistent with the results presented in this paper. In another report, the change in proton conductivity of BaCe_0.90_Y_0.10_O_3-δ_ after substituting Mn was less significant in wet hydrogen compared to air within the temperature range of 600–1000 °C.^49^ Considering that the protonic conductivity is dominant in wet reducing atmospheres at low temperatures (<500 °C), whereas mixed ionic-electronic conductivity may manifest in oxidizing atmospheres at high temperature,^13,50,51^ the results presented in this paper suggest that Mn:BZY could be a promising proton conductor operating at low temperature (300–600 °C) in wet reducing atmospheres. In real-world applications like protonic ceramic electrolysis cells (PCECs) and fuel cells (PCFCs), BZY-based electrolytes are typically co-sintered with Ni-composite electrodes, which can lead to cell performance degradation. This suggests the need for further investigation using a combinatorial approach by measuring bilayers of thin films.

The disparity between thin film and pressed pellet conductivities stems from inherent differences in the fabrication processes and microstructural characteristics of the two sample types. Thin films, produced through pulsed laser deposition (PLD), often exhibit higher conductivities compared to their bulk counterparts. This enhanced conductivity can be attributed to factors such as film crystallinity, surface defects, and reduced grain boundary resistance. In contrast, pressed pellets, typically prepared for bulk conductivity measurements, undergo a different sintering process. The pressing and sintering conditions influence grain size, porosity, and overall microstructure, contributing to variations in conductivity. The densification achieved in pressed pellets may lead to a reduction in grain boundaries, affecting proton conduction pathways and, consequently, conductivity values. It is essential to acknowledge these differences when interpreting and comparing conductivity results between thin films and pressed pellets. While thin films may demonstrate higher conductivities, the conditions and microstructural aspects associated with pressed pellets should be considered to ensure a comprehensive understanding of the material’s electrochemical behavior.

## 4 Summary and Conclusions

In conclusion, this study provides new insights into the enhanced sinterability and proton conductivity of Y-doped BaZrO_3_ (BZY) proton conductors through the incorporation of manganese (Mn) additives. Combinatorial pulsed laser deposition (PLD) was employed to synthesize BZY thin films with varying Mn concentrations (0–6%) and sintering temperatures (900–1200 °C). The synergistic combination of advanced materials synthesis techniques and rigorous characterizations has yielded several noteworthy observations.

Mn additives significantly improve the sinterability of BZY, leading to larger grain sizes and a reduction in grain boundary concentration. This enhancement is attributed to the reduced eutectic temperature of Mn-containing perovskites, which promotes partial melting at grain boundaries and facilitates grain growth. The Mn additive has a lower melting point compared to other studied additives, making them particularly effective in promoting sintering at lower temperatures. Moreover, the introduction of Mn additives results in an ideal Goldschmidt’s tolerance factor, which improves ionic conductivity and proton transport. This is in contrast to other additives, which tend to reduce the tolerance factor and hinder proton conductivity. Proton conductivity measurements reveal that BZY with Mn additives exhibits favorable performance at lower temperatures (400–500 °C) in wet hydrogen, making it a promising candidate for applications like protonic ceramic electrolysis cells (PCECs) and fuel cells (PCFCs). In light of these findings, we recommend a Mn concentration range of 2–4%, as it demonstrates substantial benefits in promoting both grain growth and enhanced electrical conductivity. The study also shows the potential of combinatorial thin film approaches for investigating mass-transport properties in oxide materials, offering a high-throughput method for future materials research.

Overall, the integration of Mn additives into BZY proton conductors represents a significant advancement in enhancing their sinterability and proton conductivity, fostering the development of superior and lower-temperature protonic ceramic electrolysis and fuel cell technologies.

## 5 Experimental Methods

Compositionally graded sample libraries of single layers of BZY and Mn-substituted BZY as well as bilayers of BZY/BHY and Mn-substituted BZY/BHY were deposited on sapphire substrates using pulsed laser deposition (PLD) at 700 °C and sintered at temperatures ranging from at 900 to 1200 °C for 2 h in air. Automated spatially resolved X-ray diffraction (XRD) and X-ray fluorescence (XRF) measurements determined crystal structure and composition on the 40-point combinatorial grid of the substrate. The resulting data was analyzed using open-source CombIgor package^52^ for Igor Pro, and is available through High-Throughput Experimental Materials (HTEM)^53^ Database. The surface morphology of selected regions of each library was examined using scanning electron microscopy (SEM) and transmission electron microscopy (TEM). Elemental distributions of the libraries were analyzed through time-of-flight secondary ion mass spectrometry (ToF-SIMS), with results analyzed using Jupyter 6.1.0. To evaluate the effect of Mn additive in BZY for the proton conduction, BZY and BZY with Mn additive powders were synthesized, pressed into pellets, and sintered at 1550 °C for 15 h, and their conductivities at intermediate temperatures (400–700 °C) in wet H_2_ (*p*H_2_O = 0.05 atm) were measured. Further experimental details are available in the Supporting Information.

## Supporting Information Available

Detailed information on additional experiments, crystal structures, particle sizes, SIMS depth profiles, and electrochemical analyses of both BZY and BZY:Mn (PDF)
