# Supplementary material for: Mn Additive Improves Zr Grain Boundary Diffusion for Sintering of a Y-Doped BaZrO3 Proton Conductor
Source: ACS Appl Mater Interfaces. 2024 Feb 22;16(9):11646–55. doi: 10.1021/acsami.3c16359 (PMC10921378; doi:10.1021/acsami.3c16359)
Supplement: Supplementary file 1 — am3c16359_si_001.pdf [file am3c16359_si_001.pdf]

Supporting Information

Mn Additive Improves Zr Grain Boundary Diffusion for Sintering of a Y-Doped $BaZrO_3$ Proton Conductor

Su Jeong Heo,[*,†,‡] Steven P. Harvey,[†] Andrew G. Norman,[†] Muhammad Anisur Rahman,[§] Prabhakar Singh[§] and Andriy Zakutayev[*,†]

[†] Materials Science Center, National Renewable Energy Laboratory, Golden, Colorado 80401, United States

[‡] Advanced Fuel Cycle Technology Development Division, Korea Atomic Energy Research Institute, 111 Daedeok−daero, Daejeon 34057, South Korea

[§] Department of Materials Science and Engineering, University of Connecticut, Storrs, Connecticut 06269, United States

[*]Email: sujeongheo@kaeri.re.kr

[*]Email: Andriy.Zakutayev@nrel.gov

Experimental Methods

1. Synthesis

The BZY/BHY and Mn-doped BZY/BHY composition libraries were deposited using pulsed laser deposition (PLD) from commercial targets with nominal compositions $BaZr_{0.8}Y_{0.2}O_3$ (BZY), $BaHf_{0.8}Y_{0.2}O_3$ (BHY), and MnO (Plasmaterials, 99.9%). The PLD instrument employed a 248 nm KrF laser with an energy density of approximately 2.6 J/cm^2 and laser pulse frequency of 20 Hz (Coherent). During the combinatorial deposition, the films were kept at a constant temperature of 700ºC and pressure of 20×10^{-3} Torr with continuous oxygen flow used to achieve total deposition pressure. The bilayer films were deposited on four sapphire substrate strips (*c*-plane, 0.5 in. × 2 in, University Wafer) to study elemental distributions through time-of-flight secondary ion mass spectrometry.

Furthermore, the single layers of BZY and Mn-, Zn-, Ni-doped BZY combinatorial films were duplicated on a 2" diameter sapphire substrate to compare their composition, crystal structures, and surface morphologies. The Zn- and Ni-doped BZY films were synthesized using PLD with commercial ZnO and NiO targets (Plasmaterials, 99.9%), and the deposition conditions were identical to those used for the single layer deposition of Mn-doped BZY film. Upon completion of the deposition, the bilayers and single layers of the PLD-deposited thin films were subsequently annealed at 900-1200°C for 2 hrs in air. The *c*-plane Al_2O_3 single crystal substrates were employed to avoid cation diffusion intake from the backside during the annealing at high temperature, but the Ba and Al interdiffusion at the BZY/substrate interface is observed that might be due to the reaction of Ba with Al which is consistent with the Ba_2AlO_4 phase appearing above 1200ºC and thus sintering at temperatures higher than 1200ºC was not performed to avoid effects of any other parameters on the diffusion studies.

2. Characterization

Structural X-ray diffraction (XRD) measurements were performed to determine the crystal structures and phase distribution of the thin films using a Bruker D8 diffractometer using θ-2θ offset scanning mode with Cu Kα radiation from a Bruker IμS source and a high resolution of two-dimensional detector. Spatially-resolved X-ray fluorescence (XRF) measurements were performed to determine the library thickness and composition using an FISHERSCOPE X-RAY XUV 773 system with an X-ray beam approximately 2 mm in diameter. Both XRF and XRD

was performed on a 4 mm spacing of 11 positions on 0.5 in x 2 in libraries and 40 points on 2 in libraries. The 40-point combinatorial grid included 4 rows with 12.5 mm spacing, as well as 9 columns ($1^{st}/4^{th}$ row) and 11 columns ($2^{nd}/3^{rd}$ row) with 4 mm spacing. The data analysis for the samples was conducted using open-source CombIgor, a data analysis package for the combinatorial experiments[1] and the resultant data is available through High Throughput Experimental Materials (HTEM)[2] Database. To obtain surface morphologies as a function of cation composition and sintering temperature, carefully selected regions of each library were examined by scanning electron microscopy (SEM) using a Hitachi S4800 equipped with energy dispersive X-ray spectrometry (EDX) system. The summary of high-throughput combinatorial experiments and analysis is presented in Figure S1. The distributions of grain size were estimated by ImageJ 1.53a software. The fin film cross-sectional morphologies and crystallinities were examined with transmission electron microscopy (TEM). The TEM micrographs were acquired with an FEI Tecnai F20 transmission electron microscope with scanning capabilities operating at an acceleration voltage of 200 kV. Specimens for the TEM were prepared by the focused ion beam (FIB) lift-out methods using a FEI Nova FIB 200 with the final Ga ion milling performed at 5kV and 81 pA and achieved a final thickness of approximately 100 nm.

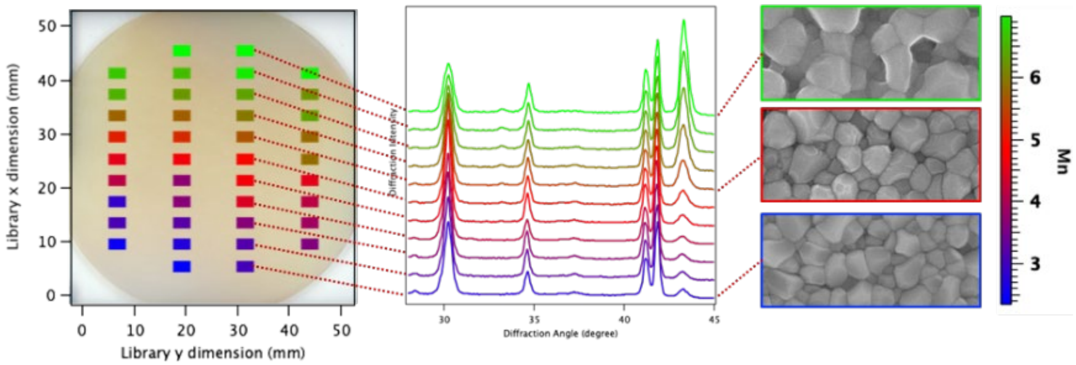

Figure S1. Combinatorial deposition on a 2-inch sapphire substrate, along with the analysis of 40 points for EDS results depicting Mn content in BZY, and selected XRD and SEM results.

3. Depth Profile Measurements and Analysis

Time-of-flight secondary ion mass spectrometry (ToF-SIMS) analysis were performed to determine elemental distributions in the bilayers of BHY|BZY and Mn-doped BHY|BZY libraries using a hybrid ION-TOF SIMS V instrument. The hybrid system is a TOF-SIMS V

basic configuration instrument (including a 30 keV three lens BiMn primary ion gun operating at 1pA pulsed beam current), with a TOF-SIMS generation IV sputter gun and a detector. A 3KeV Cs beam at 25-30nA current was used for the sputter source when profiling. TOF-SIMS profiling, imaging, and 3D tomography were used to measure the lateral and depth distribution of Mn-substituted BHY|BZY bilayers.

To determine the Zr diffusivity into the BHY layer from BZY layer, the measured depth profiles of the ^{90}Zr were normalized to the top surface and corrected by the background isotope fraction as a function of the distance. The sputter time was converted to profile depth using the thickness values of the films measured by XRF and normalized the profile depth by the subtraction of the average depth length, giving positive and negative values to distinguish the BZY and BHY layers. The normalized ^{90}Zr fraction depth profiles can be then fitted using the appropriate Crank's solution[3] to the diffusion equation for a semi-infinite medium as follow.

$$\frac{C(x,t) - C_{bg}}{C_s - C_{bg}} = A \cdot \mathrm{erf}\left(\frac{z}{2\sqrt{D * t}}\right) \qquad (1)$$

Where A is the proportional constant, D^* is the diffusion coefficient. A best fit solution to eq. (1) was determined from the least-squares method using Jupyter 6.1.0 software.

4. Diffusion mechanism modeling

In the Harrison's classification,[4] there are three different diffusion kinetics supporting bulk and grain boundary diffusion depending on diffusion time or temperature. Among them Harrison type B classified in the intermediate range of temperature and time, and the grain size *d* exceeds bulk diffusion length, $\sqrt{D_b t}$, which is attributable to both grain boundary diffusion and a bulk diffusion in a single profile. Here, the grain boundary diffusion coefficient of the BZY can be obtained using the solution provided by Chung and Wuensch,[5] which is an improved method based on Whipple's exact solution especially for analysis of SIMS depth profiles. It defined taking into account the annealing time (*t*) and bulk diffusion coefficient (D_b), and the gradient of a plot of ln$C$(x,t) against $\eta^{6/5}$ is taken.

$$D_{gb} \cdot \delta = 2D_b\sqrt{D_b t} \cdot \left[10^A\left(-\frac{\partial \ln C(x,t)}{\partial \eta^{6/5}}\right)^B\right] \qquad (2)$$

Here $\eta = x/\sqrt{D_b t}$ is a normalized depth and A and B are empirical fitting parameters that depend on the slope $\frac{\partial \ln C(x,t)}{\partial \eta^{6/5}}$ of the grain boundary tails of the TOF-SIMS depth, as tabulated in

Chung and Wuenshch.[5] Eq (2) is valid in the penetration depth range of $6 \le \eta \le 10$ in the plots of $\ln C\ \ vs. \eta^{6/5}$ as shown in Fig. S6 to determine the grain boundary diffusion coefficient.

5. Powder synthesis and electrical conductivity

5.1. Powder synthesis

$BaZr_{0.8}Y_{0.2}O_{3-\delta}$ (BZY20) were synthesized by a citrate sol-gel method for electrical conductivity evaluation. Starting materials were commercial $Ba(NO_3)_2$ (Sigma Aldrich, 99.99%), $Zr(NO_3)_2\cdot xH_2O$ (Sigma Aldrich, 99.99%), and $Y(NO_3)_3\cdot 6H_2O$ (Aldrich, 99.99%). To synthesize $BaZr_{0.8}Y_{0.2}O_{3-\delta}$ (BZY20) with 2 wt.% Mn additive powders additional $Mn(NO_3)_2\cdot 4H_2O$ powders were mixed with the starting materials in the required stoichiometry. Citric acid was used as a chelating agent. Metal cations and citric acid in 1:1 ratio were mixed in 200–300 mL of deionized water until a transparent solution was obtained. The pH was adjusted using NH_4OH (Sigma Aldrich 99.99%) to 7 to 9. Subsequently, the solution was aged at 160 °C under continuous stirring to promote gel formation. The gel was further heated at 500 °C to convert into a blackish ash. The ash was ground to powder form using mortar pestle followed by ball milling and calcined at 1100 °C for 8 h. The calcined samples were ball-milled to achieve 4.5 m^2/g BET surface area, and then, ball milled powers were pressed uniaxially into green disk pellets by hydrostatic pressing at 100 MPa. The pellets then were sintered in air at 1550 °C for 15 h for conductivity measurement.

5.2. Conductivity

Symmetrical cells were fabricated with two symmetrical porous Ag electrodes (ESL ElectroScience) were deposited onto both sides of BZY and BZY:Mn pellets and heated to 800 °C for 1 hr. Ag wires and Ag gauzes (Alfa Aesar) were used as current collectors for the electrodes. Electrochemical impedance spectroscopy (EIS) measurements were performed in humidified (～3 vol.% H_2O) 5% H_2/N_2 atmosphere using a multichannel potentiostat VMP3 (Bio-Logic Co.), in the range of 10 mHz – 200 kHz frequency with an applied ac voltage amplitude of 10 mV. ZSimpWin software was used to fit the acquired impedance data to equivalent circuits (R_bQ_b)($R_{gb}Q_{gb}$)(R_pQ_p) where R_b and R_{gb} are associated to the bulk and grain boundary ohmic resistance, and Q_{gb} is the constant phase associated to the grain boundary capacitance while R_p and Q_p are the resistance and constant phase element due to electrode processes. The bulk and grain boundary

conductivity were calculated as $\sigma_b = {}^{L}/_{AR_b}$ or $\sigma_{gb} = {}^{L}/_{A(R_b + R_{gb})}$, where L and A are the thickness and the surface area of the measured pellets, respectively.

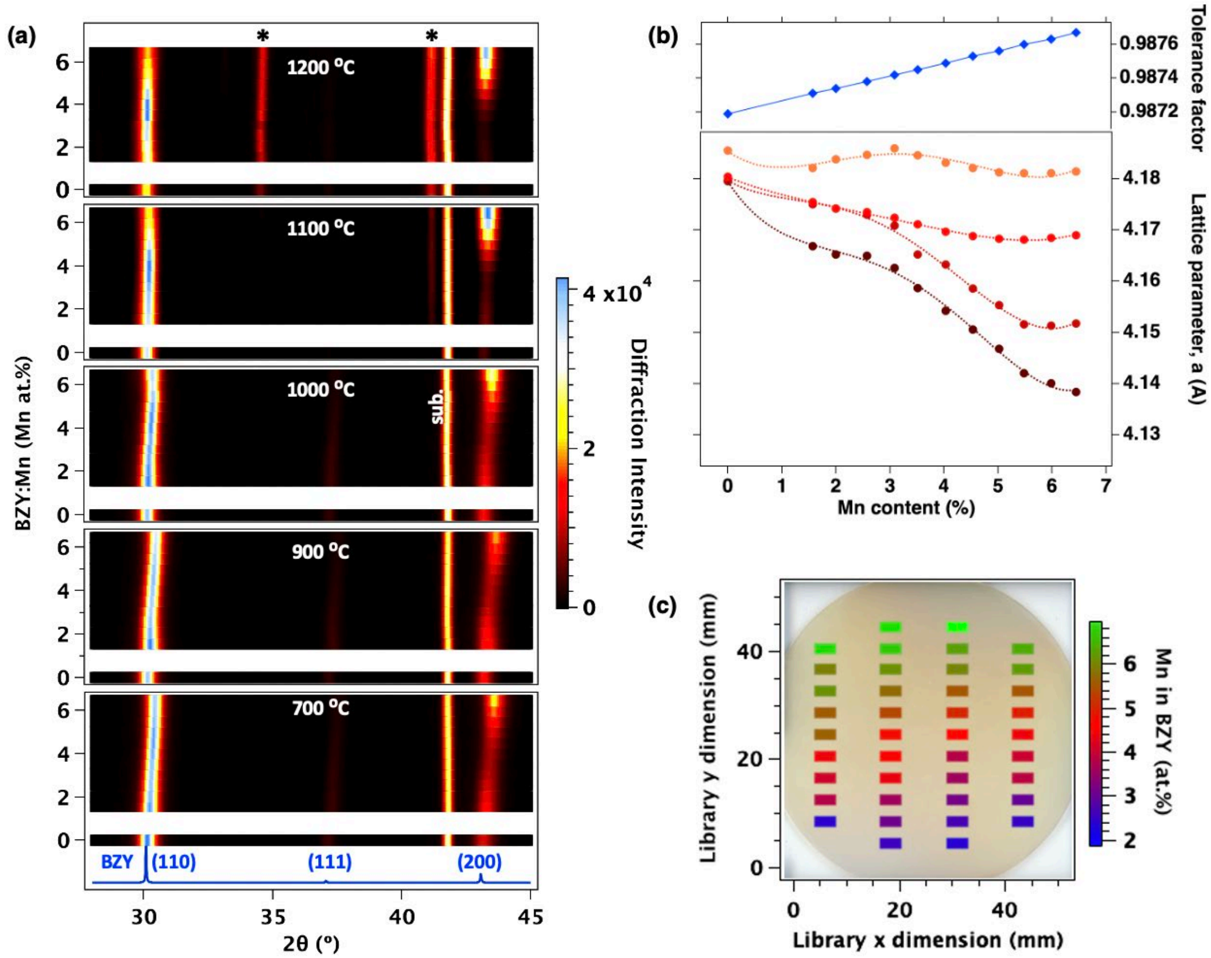

Figure S2. Crystal structure of BZY thin films as functions of Mn additive content and sintering temperature. (a) Color scale map of intensity for θ-2θ XRD measurements after sintering at 700-1200ºC. (b) Lattice parameter obtained from the XRD results and the corresponding tolerance factors. (c) Mn-content over the combinatorial Mn-substituted BZY film deposited on a sapphire substrate. Possibly $BaAl_2O_3$ phase[6] is marked with (*) label in (a).

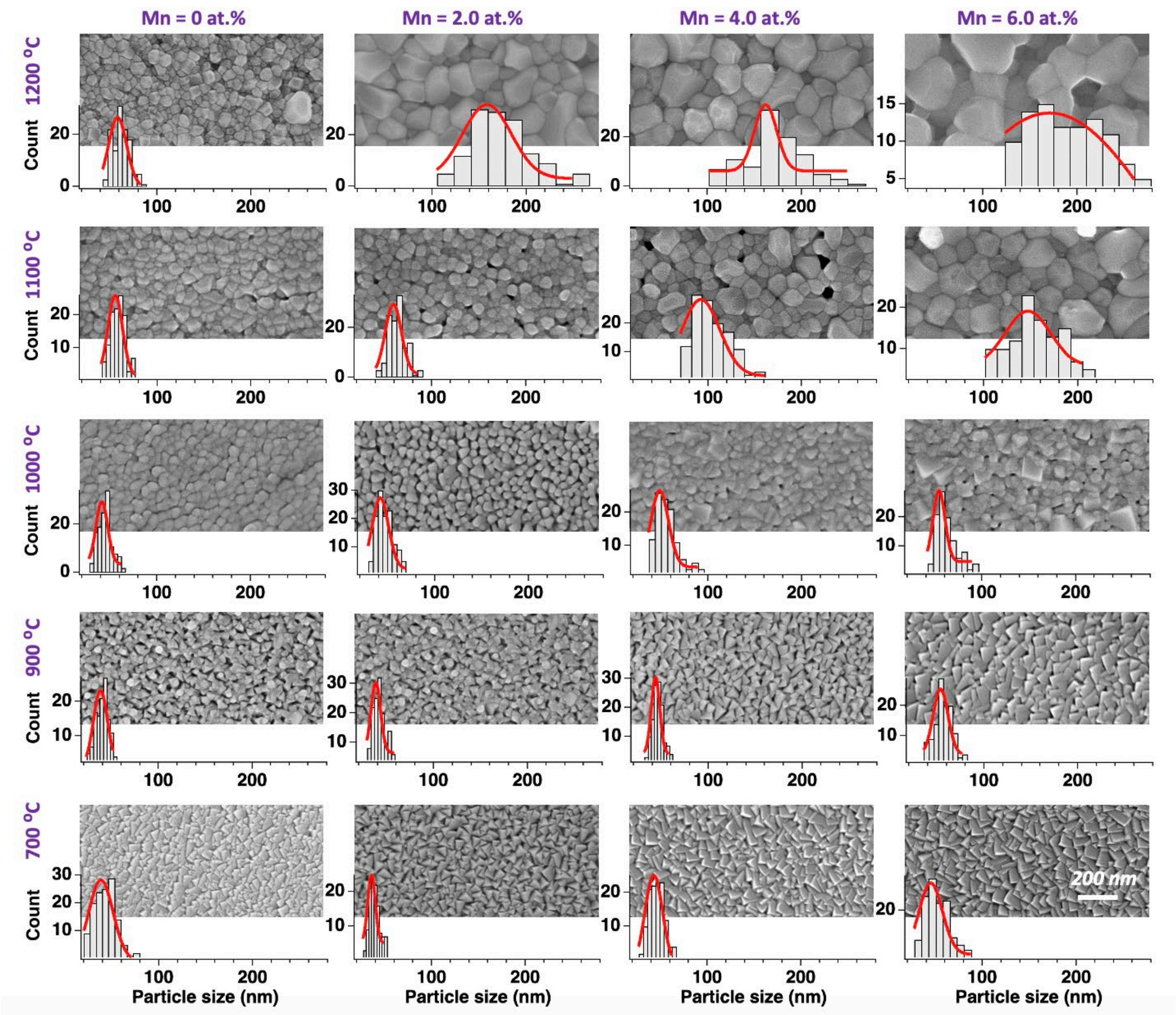

Figure S3. SEM surface micrographs of BZY and BZY:Mn (Mn = 0 – 6.0 at.%) thin films after sintering at 700 – 1200 ºC. The distributions of grain size were inserted in each SEM images.

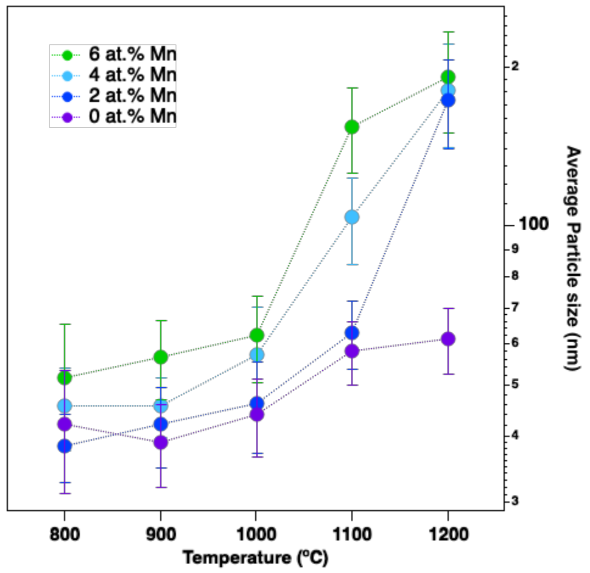

Figure S4. Average particle size dependent on Mn content and sintering temperatures obtained by the SEM images.

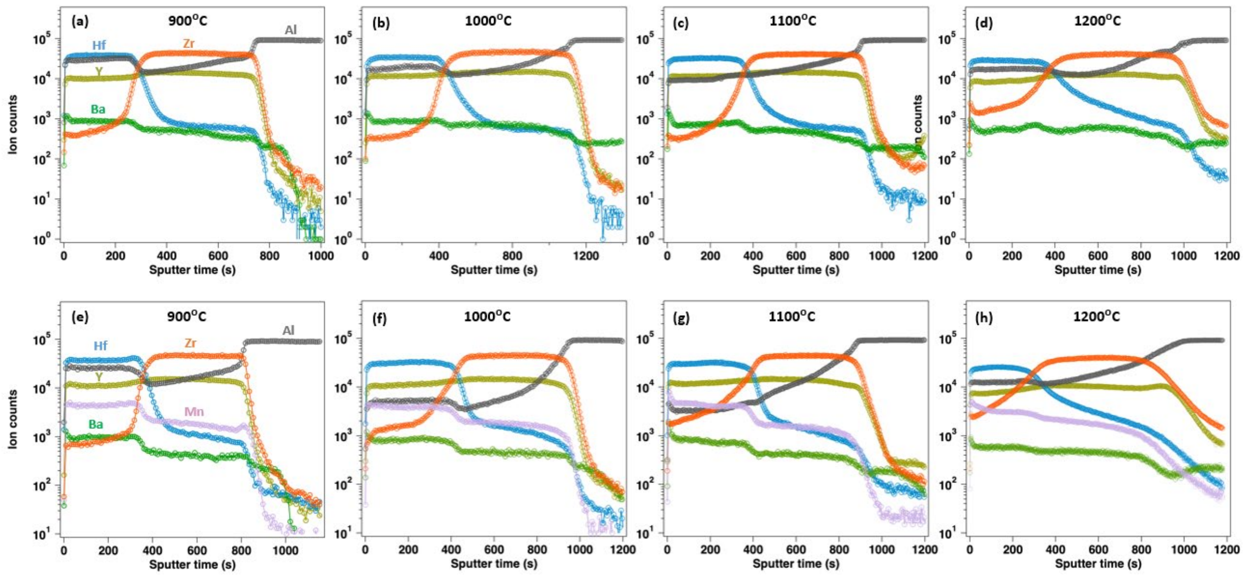

Figure S5. Typical SIMS depth profiles of the different species contained in the BHY|BZY bilayers deposited on the sapphire substrates sintered at 900-1200ºC (a-d) and BHY:Mn|BZY:Mn (Mn = 4.0 at.%) bilayers at 900-1200ºC (e-h).

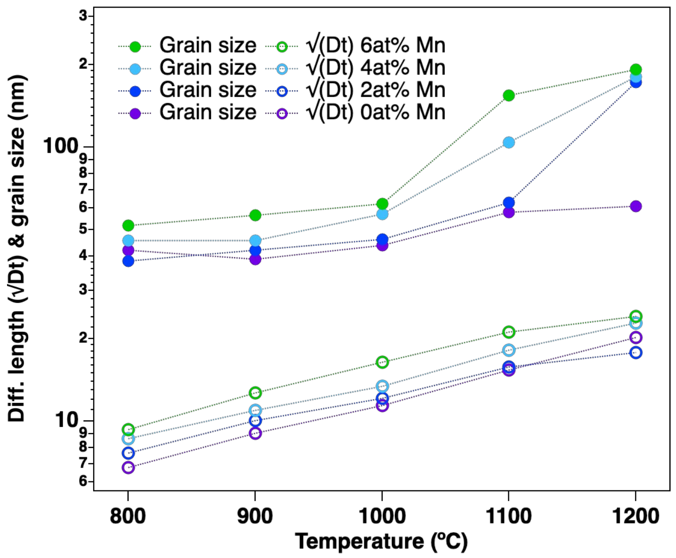

Figure S6. Distribution of grain size and bulk diffusion length $\sqrt{D_b t}$ as a function of sintering temperature in different Mn content in BZY thin films.

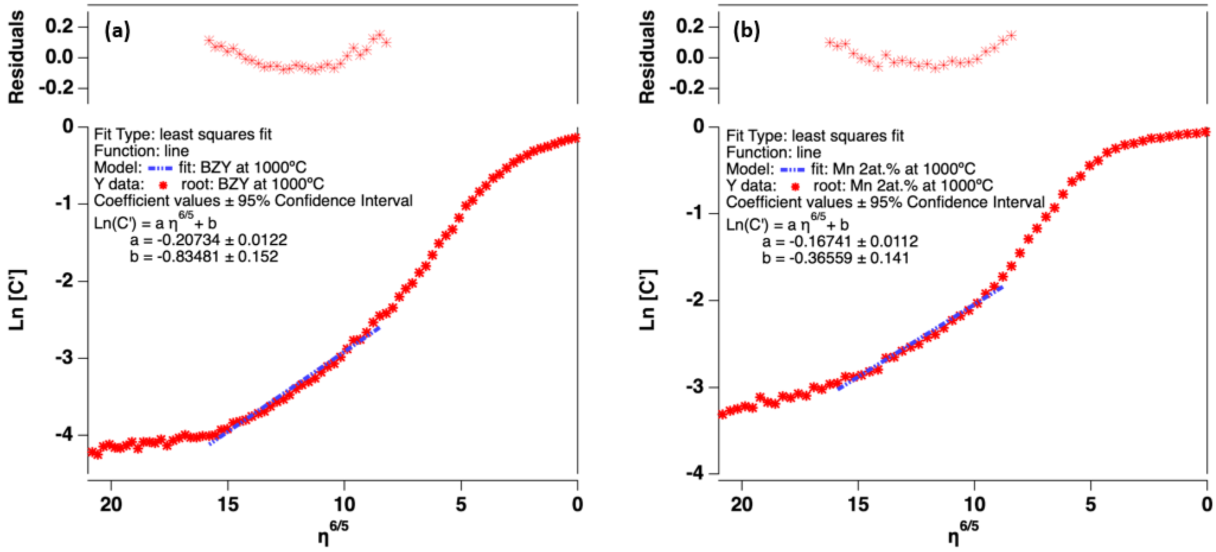

Figure S7. The normalized ^{90}Zr fraction depth profiles (Ln C') against the normalized depths ($\eta = x/\sqrt{D_b t}$) to evaluate grain boundary coefficient based on Whipple's exact solution for the bare BZY (a) and Mn-doped BZY (b) samples annealed at 1000°C. The profiles are shown together with the residuals of the fitting for the grain boundary region within the depth range $6 \leq \eta \leq 10$.

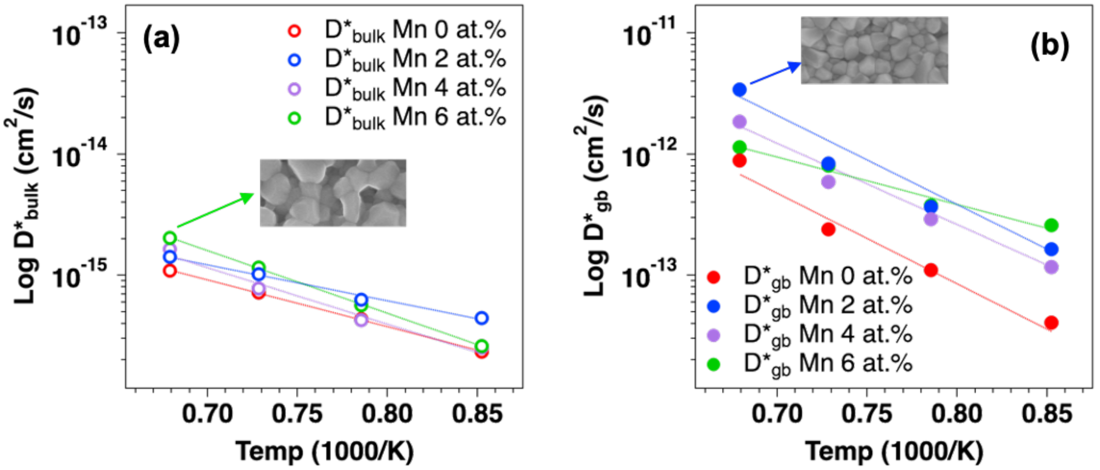

Figure S8. Cation diffusion properties of the BZY and Mn:BZY films. Arrhenius plots of the bulk (a) and grain boundary (b) diffusion coefficients obtained by the Zr concentration profiles from ToF-SIMS.

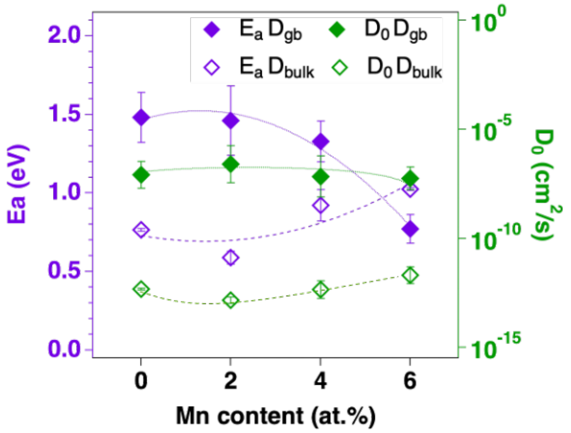

Figure S9. The activation energy (E_a) and corresponding the pre-exponential factor (D_0) for both the D_b and D_{gb} as a function of the Mn-content in BZY. The error bars represent an uncertainty due to Arrhenius fit.

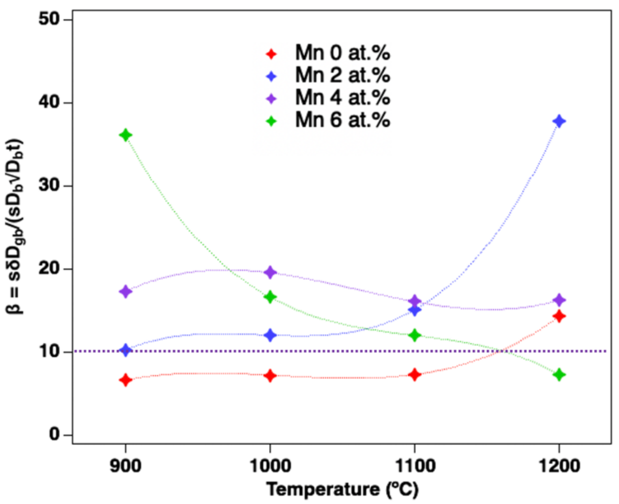

Figure S10. The critical parameter β factors to determine the ratio of the bulk and grain boundary diffusion to the profile. The β factors higher than 10 (dotted line) indicate that the grain boundary diffusivity is suitable.[7]

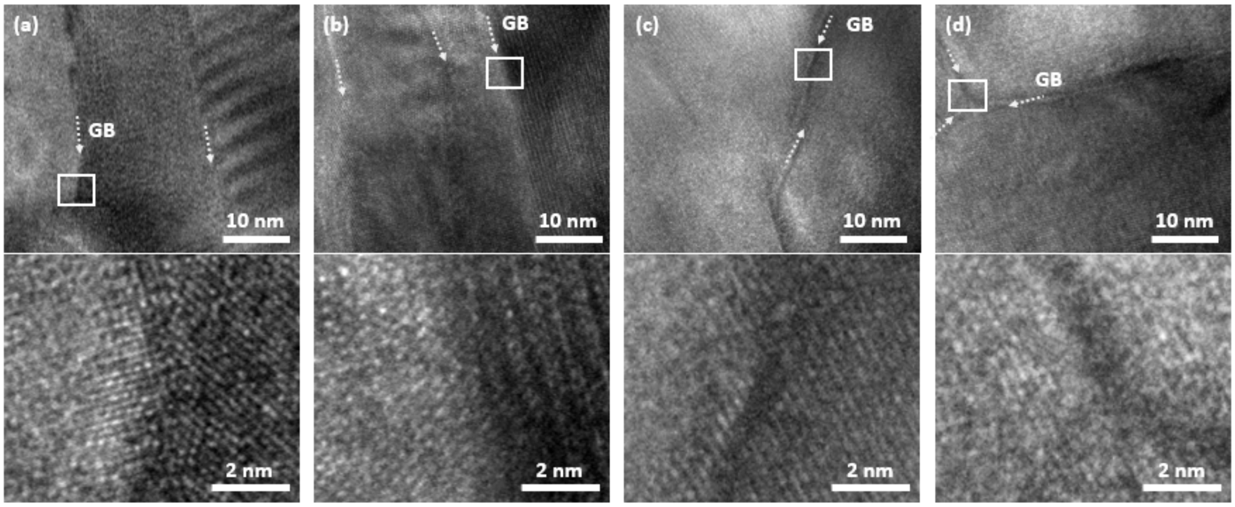

Figure S11. TEM (up) and HRTEM (below) images depicting the grain boundaries of (a, b) BZY and (c, d) BZY:Mn (Mn = 4%) films sintered at 1100ºC. HRTEM images deduced from the marked areas in upper TEM images.

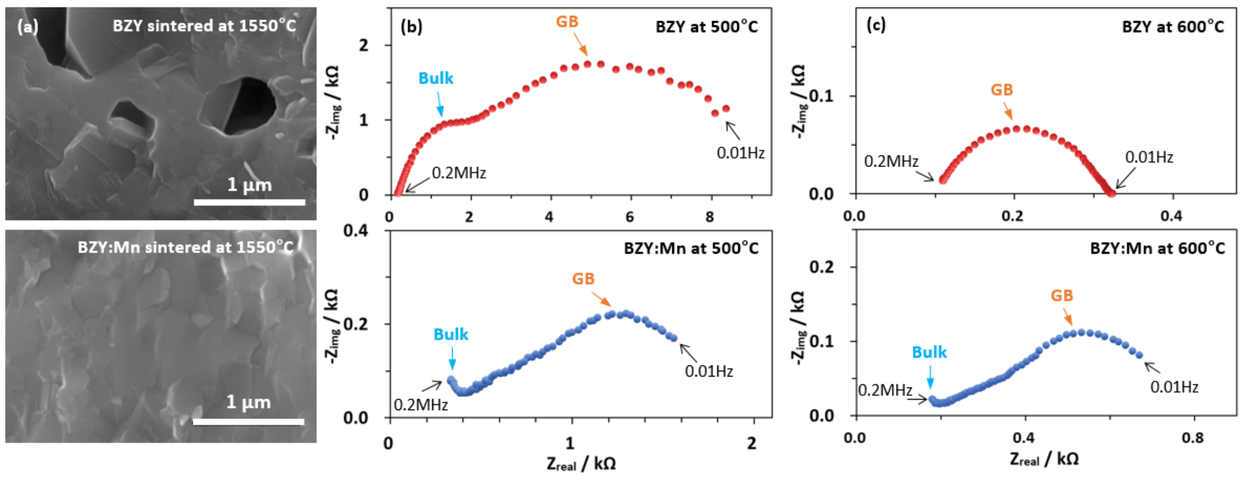

Figure S12. Microstructures and electrochemistry of BZY and BZY:Mn pellets. (a) The fracture cross section of BZY (top) exhibits the presence of pores, corresponding to the high porosity (29.7%) (not shown here). With Mn additive in BZY (bottom), even the relative low sintering temperature is sufficient to obtain densified fracture with low porosity (12.2%), implying higher density grain boundaries. Nyquist plots of BZY (top) and BZY:Mn (bot.) measured at 500ºC (b) and 600ºC (c) in wet H_2. The impedance spectra consisted of two semicircles at differing frequencies. The low frequency arc is attributed to grain boundary processes whereas the high frequency arc is attributed to the bulk response.

References

(1) Talley, K. R.; Bauers, S. R.; Melamed, C. L.; Papac, M. C.; Heinselman, K. N.; Khan, I.; Roberts, D. M.; Jacobson, V.; Mis, A.; Brennecka, G. L.; Perkins, J. D.; Zakutayev, A. COMBIgor: Data-Analysis Package for Combinatorial Materials Science. *ACS Comb. Sci.* **2019**, *21* (7), 537–547. https://doi.org/10.1021/acscombsci.9b00077.

(2) Zakutayev, A.; Wunder, N.; Schwarting, M.; Perkins, J. D.; White, R.; Munch, K.; Tumas, W.; Phillips, C. An Open Experimental Database for Exploring Inorganic Materials. *Sci. Data* **2018**, *5* (1), 180053. https://doi.org/10.1038/sdata.2018.53.

(3) Crank, J. *The Mathematics of Diffusion*, 2nd ed.; Oxford University Press: London, England, 1979.

(4) Harrison, L. G. Influence of Dislocations on Diffusion Kinetics in Solids with Particular Reference to the Alkali Halides. *Trans. Faraday Soc.* **1961**, *57*, 1191. https://doi.org/10.1039/tf9615701191.
(5) Chung, Y.-C.; Wuensch, B. J. An Improved Method, Based on Whipple's Exact Solution, for Obtaining Accurate Grain-Boundary Diffusion Coefficients from Shallow Solute Concentration Gradients. *J. Appl. Phys.* **1996**, *79* (11), 8323–8329. https://doi.org/10.1063/1.362544.
(6) Develos, K. D.; Yamasaki, H.; Sawa, A.; Nakagawa, Y. On the Origin of Surface Outgrowths in Pulsed-Laser-Deposited YBCO/CeO_2/Al2O_3 Thin Films. *Physica C Supercond.* **2001**, *361* (2), 121–129. https://doi.org/10.1016/s0921-4534(01)00297-0.
(7) Téllez, H.; Druce, J.; Hong, J.-E.; Ishihara, T.; Kilner, J. A. Accurate and Precise Measurement of Oxygen Isotopic Fractions and Diffusion Profiles by Selective Attenuation of Secondary Ions (SASI). *Anal. Chem.* **2015**, *87* (5), 2907–2915. https://doi.org/10.1021/ac504409x.
